# Molecular Identification and Characterization of a Novel Gammaherpesvirus in Wild Rabbits

**DOI:** 10.3390/v17070967

**Published:** 2025-07-10

**Authors:** Fábio A. Abade dos Santos, Ana Duarte, Inês C. Varandas, Silvia S. Barros, Ana M. Henriques, Teresa Fagulha, Margarida D. Duarte

**Affiliations:** 1 Instituto Nacional de Investigação Agrária e Veterinária, Av. da República, Quinta do Marquês, 2780-157 Oeiras, Portugalmargarida.duarte@iniav.pt (M.D.D.); 2 I-MVET, Faculdade de Medicina, Veterinária de Lisboa, Centro Universitário de Lisboa, Universidade Lusófona, Campo Grande 376, 1749-024 Lisboa, Portugal; 3 Animal and Veterinary Research Centre (CECAV), University de Trás-os-Montes e Alto Douro (UTAD), 5000-801 Vila Real, Portugal; 4 Centre for Interdisciplinary Research in Animal Health (CIISA), Faculdade de Medicina Veterinária, Universidade de Lisboa, Avenida da Universidade Técnica, 1300-477 Lisboa, Portugal

**Keywords:** herpesviruses, *Gammaherpesvirinae*, gammaherpesvirus, *Oryctolagus cuniculus algirus*, wild rabbit, Iberian rabbit

## Abstract

To date, five herpesviruses have been identified in Leporidae (LeHV-1, LeHV-2, LeHV-3, LeHV-4, and LeHV-5). Two of these have been shown to infect the European rabbit (Oryctolagus cuniculus), causing either asymptomatic infection (LeHV-2, a gammaherpesvirus) or virulent disease (LeHV-4, an alphaherpesvirus). Unfortunately, apart from LeHV-4, for which complete genome sequences are available, molecular data on leporid herpesviruses are extremely limited, with no sequences available in public databases for LeHV-1 and LeHV-3, and only a few short sequences for LeHV-2 and LeHV-5. In this study, we investigated the presence of herpesviruses in biological samples from wild rabbits (*n* = 34) found dead in the field during 2024. A pan-herpesvirus nested PCR directed to the herpesviral DNA polymerase gene was used for screening. Positive samples (*n* = 14, 41.17%) were further investigated by sequencing analysis of a longer region of the *DNA polymerase* gene, as well as the *glycoprotein B* gene and the *terminase* gene. Blastn analysis of the amplicons revealed the highest similarity to gammaherpesvirus. Phylogenetic analyses based on glycoprotein B, DNA polymerase, and concatenated amino acid sequences consistently placed the newly identified LeHV-6 in close proximity to LeHV-5. Both viruses form a well-supported clade within the *Gammaherpesvirinae*, clustering with rodent-associated herpesviruses, such as Murine herpesvirus, MuHV-4, and A. sylvaticus rhadinovirus 1. Considering the species susceptibility and the nucleotide similarities with the five previously described leporid herpesviruses, we conclude that a new rabbit gammaherpesvirus has been identified, which we propose to name LeHV-6.

## 1. Introduction

The European wild rabbit (*Oryctolagus cuniculus algirus*) plays a crucial role as a keystone species, significantly influencing plant community dynamics through its grazing habits and soil fertilization. Additionally, it serves as a vital prey species for various predators with vulnerable conservation status, including the Iberian lynx (*Lynx pardinus*) and the Spanish imperial eagle (*Aquila adalberti*) [1,2]. The decline in rabbit populations has caused notable ecosystem disruptions, leading to critical reductions in predator numbers. Consequently, conservation efforts are urgently needed, especially considering the rapid decline in native rabbit populations due to habitat loss and disease.

This scenario underscores the importance of continued surveillance and applied research to enhance our understanding of the pathogens affecting this species. Among the pathogens identified so far, viruses, particularly the myxoma and rabbit hemorrhagic viruses, have had a devastating impact on wild rabbit populations, causing severe epizootics and limiting the survivors’ ability to develop long-lasting immunity [3]. Most studies on virus-induced health issues in wild Leporidae have focused on these two viruses because of their significant impact. No other viral diseases, including those caused by herpesviruses, have been investigated until recently, despite the potential for their contribution to fatal outcomes in rabbits [4].

Herpesviruses (HVs) are enveloped viruses, measuring between 200 and 250 nm in diameter, and consisting of four distinct layers comprising a core containing linear double-stranded DNA, an icosahedral capsid with a T = 16 symmetry with approximately 125 nm in diameter, a protein-rich tegument housing multiple host- and viral-derived proteins, and an external lipid envelope embedded with various viral glycoproteins [5,6,7,8].

Herpesviruses are classified under the order *Herpesvirales*, which is divided into three families, namely, *Orthoherpesviridae*, *Alloherpesviridae*, and *Malacoherpesviridae.* The *Herpesviridae* family, which includes over 100 viruses that infect mammals, birds, and reptiles, has large genomes ranging from 125 to 290 kb.

Within the *Orthoerpesviridae* family, there are three subfamilies: *Alphaherpesvirinae*, *Betaherpesvirinae*, and *Gammaherpesvirinae*. These subfamilies are distinguished by their biological properties and phylogenetic classification. The *Gammaherpesvirinae* subfamily is further divided into seven genera: *Bossavirus*, Lymphocryptovirus, Macavirus, Matnicavirus, Patagivirus, Percavirus, and Rhadinovirus.

Members of the *Alphaherpesvirinae* subfamily are known for rapid lysis in cell cultures, while *Betaherpesvirinae* members grow slowly, often leading to the formation of giant cells. *Gammaherpesvirinae* viruses primarily infect lymphoid tissues and have a tropism for lymphoid lineage cells, which can result in lymphoproliferative diseases and oncogenesis [5,6,7,8].

To date, five herpesviruses have been identified in leporids: Leporid herpesvirus 1 (LeHV-1), Leporid herpesvirus 2 (LeHV-2), Leporid herpesvirus 3 (LeHV-3), Leporid herpesvirus 4 (LeHV-4), and Leporid herpesvirus 5 (LeHV-5) (reviewed in [9]). LeHV-1, LeHV-2, LeHV-3, and LeHV-5 belong to the *Gammaherpesvirinae* subfamily, with LeHV-1 and LeHV-3 having been reported in *Sylvilagus floridanus*; LeHV-2 in both *Sylvilagus floridanus* and *Oryctolagus cuniculus*, the latter considered the natural host, where it causes asymptomatic infections; and LeHV-5 in *Lepus granatensis*. LeHV-4 belongs to the *Alphaherpesvirinae* subfamily, having also been reported in European rabbits. LeHV-2 and LeHV-3 are the most commonly identified herpesvirus infections in rabbit species (*Sylvilagus* and *Oryctolagus*) (reviewed by [9]).

Except for LeHV-5, for which virus isolation attempts failed when the virus was described, the characterization of these viruses has primarily relied on successful virus isolation or detection in cell culture. However, in terms of genetic information on these viruses, apart from LeHV-4, for which complete genome sequences are available, molecular data on rabbit herpesviruses are very scarce, with none (LeHV-1 and LeHV-3) or only a few short sequences (LeHV-2 and LeHV-5) currently available in public databases.

The impact of these herpesviruses on European rabbits varies widely, with LeHV-2 infections often going unnoticed [10], whereas LeHV-4 is much more virulent, leading to fatal infections [9]. The focus of this study was to investigate the presence of herpesvirus in wild rabbit samples by molecular methods and virus isolation.

## 2. Materials and Methods

### 2.1. Sample

This study analyzed a total of 34 wild rabbits that were found dead in the field in the center and south of mainland Portugal, specifically in the districts of Castelo Branco, Coimbra, Santarém, Mértola, Barrancos, and Castro Verde.

The rabbits were collected between February and May 2024 as part of the LIFE Iberconejo project, with no animals being sacrificed for this study. The carcasses were in acceptable post-mortem condition (estimated 2–3 days after death), as prospective monitoring enabled the prompt recovery of dead rabbits from the field.

### 2.2. Virological Examination

For nucleic acid extraction, fresh samples of the liver, spleen, lung, and kidney were collected during necropsies, which were carried out following standard guidelines.

Tissues were homogenized at 20% with phosphate-buffered saline (PBS) and clarified at 3000× *g* for 5 min. Total nucleic acid was extracted from 200 μL of the clarified supernatants, using a MagAttract 96 cador Pathogen Kit in a BioSprint 96 nucleic acid extractor (Qiagen, Hilden, Germany), according to the manufacturer’s protocol.

All the animals were tested for the presence of herpesvirus DNA using the generalist nested PCR described by Van Devanter et al. (1996) [10] that allows the detection and amplification of a *DNA polymerase* gene fragment of different herpesvirus subfamily members. Amplification of the partial *DNA polymerase* and *glycoprotein B* (*gB*) genes was performed using the systems described by Devanter et al., Ehlers et al., and Ehlers et al. [10,11,12], with the conditions previously described by Abade dos Santos et al. [9].

Partial amplification of the herpesvirus terminase gene was attempted with several primer systems, as described previously [13,14,15], and with multiple de novo-designed primer sets. Available gammaherpesvirus terminase gene sequences were aligned to design eight primer combinations intended to maximize coverage of the genetic diversity across the Gammaherpesvirinae subfamily (Table 1).

Despite extensive optimization efforts, including the use of touchdown PCR protocols, adjustments in annealing temperatures (±5 °C), varying magnesium ion concentrations, and testing different DNA polymerase enzyme systems (NZYProof and Hot Start Taq), amplification consistently failed to yield specific products. Both first-round and nested PCR strategies were applied with no success.

In general, PCR reactions were performed with NZYProof 2× Green Master Mix (Nzytech, Lisboa, Portugal) under the manufacturer’s recommended conditions using 1 μM of each primer and 50 ng of total DNA. Amplifications were carried out in a Bio-Rad CFX96™ Thermal Cycler (Bio-Rad Laboratories Srl, Redmond, WA, USA). For the systems already published, the described annealing temperatures were used in parallel with a 3 to 5 °C lower ramp temperature than the average melting temperature of the primers.

### 2.3. Sequencing Analysis

PCR products were observed in a 1.5% horizontal electrophoresis agarose gel, excised, purified with the NZYGelpure kit (Nzytech, Lisboa, Portugal), and directly sequenced using an ABI Prism BigDye Terminator v3.1 Cycle sequencing kit on a 3500 Genetic Analyzer (Applied Biosystems, Foster City, CA, USA). Nucleotide sequences were analyzed and assembled into consensus sequences using BioEdit version 7.2.5 software and submitted to GenBank. Nucleotide sequences were translated using Mega X v10.1 software. A pairwise amino acid distance matrix was calculated based on the alignment of the concatenated sequences used for phylogenetic analysis. The resulting matrix was visualized as a heatmap to assess the relative similarity between viral taxa. The heatmap was manually generated in Python 3.13.5 using the Seaborn data visualization library (v0.11.2), which offers a high-level interface for drawing attractive and informative statistical graphics. Data pre-processing and matrix formatting were performed using the Pandas library, and the color scheme was based on a continuous gradient from low (purple) to high (yellow) identity. The figure was finalized in high resolution using Matplotlib 3.10.3.

### 2.4. Phylogenetic Analysis

Partial amino acid sequences of the viral DNA polymerase and glycoprotein B (gB) were aligned using MUSCLE, with a gap opening penalty of −2.90, a gap extension penalty of 0, and a hydrophobicity multiplier of 1.2. Phylogenetic analyses were conducted using MEGA11 [17], based on the evolutionary model selected by the Model function implemented in the software.

The evolutionary history was inferred using the Maximum Likelihood (ML) method and the Le and Gascuel (LG) model, with rates among sites modeled using a discrete Gamma distribution (+G) and allowing for a proportion of invariant sites (+I). A discrete Gamma distribution with five categories was used to model evolutionary rate differences among sites, with shape parameters of 0.9049 for the gB tree, 0.6396 for the polymerase tree, and 0.8347 for the concatenated tree. The rate variation model allowed for a proportion of invariant sites of 8.02%, 16.99%, and 10.31%, respectively.

Initial tree(s) for the heuristic search were obtained automatically by applying the Neighbor-Join and BioNJ algorithms to a matrix of pairwise distances estimated using the JTT model, selecting the topology with the superior log-likelihood value. The bootstrap consensus tree was generated from 1000 replicates to represent the evolutionary history of the taxa analyzed. Branches reproduced in less than 70% bootstrap replicates were collapsed, and the percentage of replicate trees in which the associated taxa clustered together was shown next to the branches.

All positions with less than 95% site coverage were eliminated from the alignments, meaning positions with more than 5% alignment gaps, missing data, or ambiguous bases were removed (partial deletion option). The final datasets contained 165 positions for the gB tree, 60 positions for the polymerase tree, and 225 positions for the concatenated tree, comprising sequences from 51 taxa in each case.

Pairwise amino acid identities were calculated based on the concatenated gB and polymerase sequences, and a heatmap was generated to visualize the identity matrix [17].

### 2.5. Virus Isolation

The isolation of herpesvirus was attempted from the organs of four rabbits positive for herpesvirus and negative for MYXV and six rabbits positive for herpesvirus and positive for MYXV, namely, from the liver, spleen, lung, and kidney, in BSL-2 conditions.

Samples were homogenized at 20% (*w*/*v*) in phosphate-buffered saline containing penicillin, streptomycin, and amphotericin B (antibiotic–antimycotic), used according to the manufacturer (Gibco, Life Technologies Corporation, CA, USA). Following centrifugation, the supernatant was filtered through a 0.45 μm pore size filter (Millipore Express, Billerica, MA, USA) and used to inoculate sub 70% confluent Vero cells (ATCC No. CRL-1986), Rabbit Kidney (RK13) cells (ATCC-CCL-37), the BJAB cell line (ACC 757), and wild rabbit primary cells [18].

The RK13 cells were grown in Eagle medium, and all the other cells were grown in Dulbecco’s modified Eagle’s Media, supplemented with 10% fetal calf serum (Gibco), penicillin, streptomycin, and amphotericin B (antibiotic–antimycotic used at 1:100), and 50 μg/mL gentamicin (Gibco). The cells were maintained at 37 °C under a humidified atmosphere with 5% CO_2_ and observed daily for the cytopathic effect (CPE) by phase contrast microscopy. Three blind passages were carried out. The supernatant was tested by PCR for herpesvirus DNA presence. In the case of the samples positive for MYXV, they were neutralized by incubation with an anti-MYXV antibody cocktail for 2 h at 37 °C prior to cell inoculation.

## 3. Results

### 3.1. Necropsy and Histopathological Findings

No clinical lesions typically associated with herpesvirus infection were observed in any of the examined rabbits. The sample set comprised animals that either presented no detectable lesions at necropsy or exhibited lesions characteristic of myxomatosis, which may have potentially masked subtle lesions attributable to herpesvirus infection. Histopathological analysis did not reveal any evidence of epithelial hyperplasia, vesicular lesions, or intranuclear inclusion bodies that are classically indicative of active herpesvirus replication. Similarly, no lymphoproliferative changes or other microscopic alterations suggestive of herpesvirus-associated pathology were detected in the examined tissues.

### 3.2. Virologic Screening

Conventional PCR (Van Devanter et al., 1996) detected herpesvirus-DNA in the liver, spleen, and lung samples of 41.17% (14/34) of the analyzed wild rabbits [10]. The presence of the virus was then confirmed by sequencing analysis of the DNA polymerase amplicons of all PCR-positive samples, allowing the recovery of sequences of approximately 171 bp. A subsample was subjected to additional amplification, yielding DNA polymerase and gB gene fragments of 368 bp and 605 bp, respectively.

The longest DNA fragments were typically achieved by performing an initial PCR using the described protocol, followed by a second PCR, in which the primers from both nested steps were combined. Furthermore, 4 of the 14 herpesvirus-positive samples were subjected to multiple overlapping sequencing reactions to ensure the accuracy and reliability of the assembled fragments.

The obtained nucleotide sequences were submitted to GenBank using the BankIt (PV730057 to PV730064). The four DNA polymerase sequences and four gB sequences were identical, indicating a high degree of conservation among the circulating viral strains. NCBI blast analysis (8 June 2025) of the DNA polymerase partial nucleotide sequences obtained confirmed homology with the herpesvirus DNA polymerase coding sequence from other mammals.

The top two hits of the DNA polymerase sequence BLASTn results corresponded to sequences annotated as Leporid gammaherpesvirus 5 (GenBank accession: MN514243 and MN557129), with a max score of 175 and a percent identity of 79.89 to 80.41%. These values indicate that the strain investigated in this study shares a moderate to high degree of similarity with LeHV-5, particularly within conserved regions of the DNA polymerase gene. Comparison with the available 162 bp of DNA Polymerase of Leporid gammaherpesvirus 2 (U63468.1) resulted in a query cover of 11% with 78.05% of homology.

The BLASTn results for the DNA polymerase gene revealed E-values of 9 × 10^−39^ to 3 × 10^−32^ for Leporid gammaherpesvirus 5. These extremely low E-values indicate that the observed sequence alignments are highly significant and unlikely to have occurred by random chance. Therefore, the E-values obtained confirm that the virus here identified shares meaningful homology with LeHV-5, particularly in the conserved DNA polymerase region, supporting its placement within the Gammaherpesvirinae subfamily.

Regarding the partial sequence of the gB gene, the highest-scoring alignment corresponds also to Leporid gammaherpesvirus 5 isolate, showing a max score of 550 and percent identity of 86.95%. This result, in complementarity to the results obtained with the DNA polymerase gene, indicates a strong sequence similarity between LeHV-6 and LeHV-5.

Additional notable hits include sequences from various murine and wood mouse herpesviruses (e.g., wood mouse herpesvirus, Murine herpesvirus strain 72, Murid gammaherpesvirus 4), with max scores ranging from 242 to 292 and percent identity between 68.44% and 71.40%.

The sequences also showed similarity to Human gammaherpesvirus 4 (Epstein–Barr virus), with multiple entries showing max scores between 67.1 and 71.6, query cover values ranging from 52% to 56%, and percent identity consistently around 68.39% to 71.59%. The best matches included several partial and full genome entries of EBV from different isolates and chromosomes, reflecting redundancy and database richness for this virus. Additionally, Leporid gammaherpesvirus 5 (LeHV-5), the closest known lagomorph herpesvirus, was also identified among the top results, supporting its phylogenetic proximity.

At the nucleotide level, LeHV-6 shares 83.7% (103/123) identity with LeHV-5 in the DNA polymerase gene and 87.6% (383/437) in the gB gene. The corresponding amino acid identities are 82.4% (47/57) for DNA polymerase and 97.4% (147/151) for gB, further supporting that, although closely related, LeHV-6 is genetically distinct from LeHV-5.

In summary, the BLASTn data reinforce that the virus here identified shares the highest homology with LeHV-5, but with sufficient divergence (identity < 90%) to suggest it is genetically distinct. The presence of conserved motifs in alignment with other gammaherpesviruses further reinforces its classification within the Gammaherpesvirinae subfamily, while also highlighting its distinct genetic features that set it apart from known leporid herpesviruses.

Amplification of the terminase gene was not successful despite several attempts using various systems and primers (Table 1).

### 3.3. Phylogenetic Analysis

**Figure 1:**
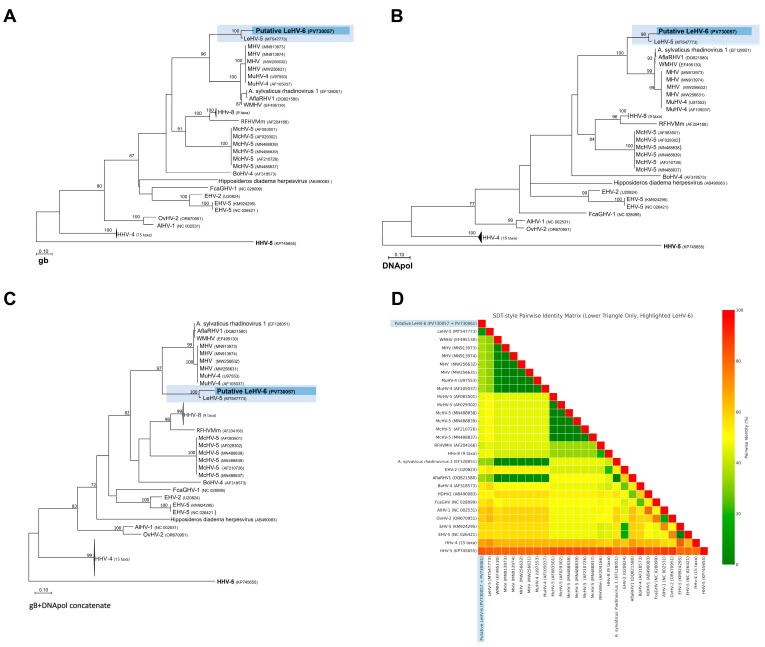
Phylogenetic analysis of the novel Leporid gammaherpesvirus 6 (LeHV-6) and representative gammaherpesviruses. (**A**) Maximum Likelihood phylogenetic tree based on the partial gB protein sequence (165 aa). (**B**) Maximum Likelihood phylogenetic tree based on the partial DNA polymerase protein sequence (60 aa). (**C**) Maximum Likelihood phylogenetic tree based on the concatenated gB and polymerase partial sequences (225 aa). (**D**) Heatmap showing pairwise amino acid identity percentages based on the concatenated sequences. Accession numbers of the sequences used are indicated in the trees are drawn to scale, with branch lengths measured in the number of substitutions per site.

Despite the relatively short length of the amplified regions, the partial amino acid sequences encoded by the gB (Figure 1A) and DNA polymerase (Figure 1B) genes proved to be sufficiently informative to infer phylogenetic relationships within the Orthoherpesviridae family. Separate and concatenated alignments of these two gene regions were used to construct phylogenetic trees by Maximum Likelihood (ML) using the LG+G+I substitution model.

In the resulting separate phylogenetic trees, the putative Leporid gammaherpesvirus 6 (LeHV-6) consistently grouped within the Gammaherpesvirinae subfamily. More specifically, LeHV-6 clustered closely with Leporid gammaherpesvirus 5 (LeHV-5), forming a strongly supported monophyletic clade (bootstrap = 100) that is phylogenetically proximal to rodent-associated rhadinoviruses, including Murine herpesvirus 4 (MuHV-4), Murine herpesvirus 68 (MHV-68), and Apodemus sylvaticus rhadinovirus 1.

When using concatenated gB and DNA polymerase sequences (Figure 1C,D), the topology of the tree improved, providing stronger resolution and higher bootstrap values for the nodes. The putative LeHV-6 remained within the Rhadinovirus clade, yet it diverged from known rodent and primate herpesviruses, reinforcing its unique position. The pairwise amino acid distance matrix, visualized through a heatmap, also supported these findings, indicating that LeHV-6 shares the highest similarity with LeHV-5 but displays clear divergence from other gammaherpesviruses.

Altogether, these phylogenetic results support the classification of LeHV-6 as a novel member of the Gammaherpesvirinae subfamily, closely related but genetically distinct from previously described leporid herpesviruses, particularly LeHV-5.

Just like Leporid gammaherpesvirus 5 (LeHV-5), the putative LeHV-6 clustered within the Rhadinovirus clade in all constructed trees. This topological pattern was consistently maintained across all datasets analyzed, suggesting that the observed phylogenetic signal is robust and not an artifact of sequence length or alignment bias.

Figure 1A shows the phylogenetic tree based on the partial gB protein sequence (165 aa). The phylogenetic analysis, conducted in MEGA11 [19], included 51 amino acid sequences and 165 positions after removing sites with <95% coverage. Evolutionary rate variation was modeled using a discrete Gamma distribution (+G, parameter = 0.9049) and a proportion of invariable sites (+I, 8.02%).

Figure 1B shows the phylogenetic tree based on partial polymerase protein sequences (60 aa). The analysis, performed in MEGA11 [20], included 51 amino acid sequences and 60 positions after removing sites with <95% coverage. Evolutionary rates were modeled using a discrete Gamma distribution (+G, parameter = 0.6396) and a proportion of invariable sites (+I, 16.99%).

Figure 1C shows the phylogenetic tree based on the partial gB protein and the polymerase concatenated sequence (225 aa). A discrete Gamma distribution was used to model evolutionary rate differences among sites (five categories (+G, parameter = 0.8347)). This analysis, conducted in MEGA11, included 51 amino acid sequences and 225 positions after removing sites with <95% coverage. The model accounted for rate variation with a proportion of invariable sites (+I, 10.31%).

Figure 1D. shows a heatmap of the concatenated protein sequences with pairwise identity. The accession numbers of the sequences used are found in the figures [9].

While additional genomic information is needed to achieve a better understanding of this virus, this preliminary analysis suggests that it may represent a distinct replicating lineage within the *Rhadinovirus* genus, together with the previously described Leporid gammaherpesvirus 5 [9].

In accordance, we propose to name this virus Leporid gammaherpesvirus 6 (LeHV-6), following the rabbit alphaherpesvirus 4 (LeHV-4) and leporid gammaherpesvirus 6 (LeHV-5).

### 3.4. Virus Isolation

It was not possible to obtain herpesvirus PCR-positive cell cultures, including the episome form or even the lytic replication form, after lytic cycle induction. This result is consistent with that previously described for Leporid gammaherpesvirus 5, which is also not isolable in cells [9].

## 4. Discussion

The study of wildlife pathogens is an essential component of biodiversity conservation, particularly in species under severe demographic pressure. In Mediterranean ecosystems, the European wild rabbit (*Oryctolagus cuniculus algirus*) plays a keystone ecological role, serving both as a major herbivore and as the primary prey for endangered predators, such as the Iberian lynx and the Spanish imperial eagle. Yet, over the last decades, this species has suffered substantial population declines, driven largely by disease outbreaks, most notably those caused by Myxoma virus (MYXV) and rabbit hemorrhagic disease virus 2 (RHDV2) [21,22,23,24,25,26]. These known viral agents have shaped much of the surveillance focus, overshadowing other potentially important pathogens such as herpesviruses.

Herpesviruses, especially members of the subfamily *Gammaherpesvirinae*, are characterized by their capacity to establish lifelong latent infections, reactivating under conditions of immunosuppression or stress [8]. Despite their ubiquity and evolutionary complexity, gammaherpesviruses in lagomorphs remain poorly described. Until now, only a handful of leporid herpesviruses had been reported, and molecular information on most of them is extremely limited or entirely lacking. This highlights a substantial gap in our understanding of leporid virome diversity and evolution.

To date, two herpesviruses have been described in the European rabbit, namely, LeHV-2, a gammaherpesvirus that causes asymptomatic infections, and LeHV-4, an alphaherpesvirus that induces severe neurological signs.

European rabbits are not susceptible to LeHV-1, LeHV-3, which infects *Sylvilagus floridanus*, or to LeHV-5, which infects Iberian hares but not *Oryctolagus cuniculus* (results not published), thus ruling out the possibility that the virus detected could be LeHV-1, LeHV-3, or LeHV-5. A sequence comparison with public database entries (available for LeHV-5, but lacking for LeHV-1 and LeHV-3) confirmed this conclusion.

For LeHV-2, although only a 162 bp *DNA polymerase* gene sequence (accession U63468) and a bp-long *terminase* gene sequence (AF091069.1) are publicly available, comparison with the sequences obtained in this study allowed us to exclude any genetic relationship with LeHV-2. Degenerated primers designed specifically for the LeHV-2 *terminase* gene did not amplify any product in LeHV-6, showing that there are significant differences in the genomic region.

Within this context, the present study identifies and characterizes a novel gammaherpesvirus infecting wild *O. cuniculus algirus*, provisionally designated as Leporid gammaherpesvirus 6 (LeHV-6). Molecular analysis based on partial sequences of the DNA polymerase and glycoprotein B (gB) genes reveals that this virus shares the highest sequence identity (~81% in DNA polymerase, ~87% in gB) with LeHV-5, a previously described gammaherpesvirus in Iberian hares (*Lepus granatensis*) [9]. These identity values fall below the thresholds typically associated with conspecific strains based on conserved genes [12,13,14], supporting the designation of LeHV-6 as a distinct viral entity, particularly because it infects a different host species than LeHV-5.

Phylogenetic analysis further corroborates this taxonomic distinction. LeHV-6 clusters tightly with LeHV-5 within the *Rhadinovirus* genus of *Gammaherpesvirinae* family, forming a robust and well-supported clade distinct from rodent and primate rhadinoviruses. The maintenance of this topological pattern across analyses using gB, DNA polymerase, and concatenated sequences suggests a stable phylogenetic signal. This also raises intriguing evolutionary questions regarding the origin and divergence of leporid rhadinoviruses, possibly reflecting ancient cospeciation events with lagomorph hosts, as suggested for other mammalian gammaherpesviruses [12].

This distinction is further supported by the failure to amplify the terminase gene of LeHV-6 despite extensive attempts using both previously published primers and eight newly designed primer pairs specifically targeting conserved regions across the Gammaherpesvirinae. In contrast, the same primer sets successfully amplified the terminase gene of LeHV-5 from Iberian hares demonstrating that the failure was not due to technical limitations but likely reflects a true genetic divergence in this genomic region. This provides additional molecular evidence that LeHV-6 and LeHV-5 represent distinct viral species.

This outcome likely results from a combination of factors, including (i) the overall low viral DNA load present in field-collected tissues, (ii) significant sequence divergence in the terminase gene of LeHV-6 relative to other gammaherpesviruses, and (iii) the inherent difficulty of amplifying non-structural genes, which tend to be more variable than highly conserved structural genes like DNA polymerase or glycoprotein B. This limitation is commonly reported in wildlife herpesvirus studies, where primer–template mismatches due to genetic divergence can severely compromise PCR success.

The failure to isolate infectious virus in cell culture mirrors findings with LeHV-5 and many other gammaherpesviruses, which are notoriously difficult to cultivate ex vivo because of their preference for lymphoid tissues and latent replication strategies [27]. This limitation reinforces the importance of molecular surveillance tools and highlights the need for novel in vitro models, such as primary rabbit lymphoid or fibroblast cell cultures, to study latent viral infections in lagomorphs [18].

Although no herpetic lesions were detected in LeHV-6-positive animals, the detection of viral DNA in lung, genitalia, and skin tissues suggests the possibility of systemic infection and/or latency outside lymphoid reservoirs. These findings are consistent with the biology of other rhadinoviruses, such as Kaposi’s sarcoma-associated herpesvirus (KSHV), which exhibit mucocutaneous and lymphotropic tropism [28].

The presence of LeHV-6 in rabbits co-infected with MYXV further raises the hypothesis of immunosuppression-facilitated viral reactivation, as has been proposed for LeHV-5 in Iberian hares [9]. These interactions, though not directly demonstrated here, are plausible and should be explored through longitudinal studies and co-infection models.

A further concern lies in the potential immunomodulatory and oncogenic properties of LeHV-6. Many gammaherpesviruses encode genes that influence the host cell cycle and apoptosis, such as vBCL-2, LANA, and viral cyclins [28]. While no neoplastic lesions were observed in this study, full genomic sequencing of LeHV-6 will be essential to evaluate the presence of such virulence determinants.

The absence of macroscopic lesions or specific pathological signs in wild rabbits infected with LeHV-6 is consistent with the biological characteristics of Gammaherpesvirinae viruses. Members of this subfamily are well known for their ability to establish lifelong latent infections in lymphoid tissues, often persisting asymptomatically in their natural hosts for extended periods [27]. Unlike alphaherpesviruses, which typically cause acute cytolytic infections associated with overt epithelial lesions, gammaherpesviruses primarily exhibit lymphotropism, targeting B or T lymphocytes, with a latent phase that predominates over lytic replication [7].

In many wildlife species, gammaherpesvirus infections are subclinical or asymptomatic, and clinical disease generally arises only under conditions that disrupt immune homeostasis, such as severe stress, immunosuppression, or coinfections [7,12]. This pattern is well documented in other host–virus systems, including Epstein–Barr virus (EBV) in humans and Murine herpesvirus 4 (MuHV-4) in rodents, where infections typically remain inapparent unless the immune system is compromised [29]. Similarly, the previously described LeHV-5 in Iberian hares was frequently detected in multiple tissues without associated lesions under normal conditions [9].

A notable difference, however, emerges when comparing the outcomes of LeHV-6 infection in rabbits with LeHV-5 infection in Iberian hares. While LeHV-6 was not associated with visible lesions even in animals co-infected with Myxoma virus (MYXV), LeHV-5 caused severe mucocutaneous lesions in hares, particularly during coinfection with ha-MYXV [9]. This discrepancy is likely explained by the distinct host–virus evolutionary dynamics. European rabbits (*Oryctolagus cuniculus*) have coexisted with MYXV for over 70 years since its introduction into Europe, leading to a co-evolutionary balance characterized by partial resistance in the host and attenuation of MYXV virulence [30]. Consequently, the level of immunosuppression induced by MYXV in rabbits is likely insufficient to trigger uncontrolled herpesvirus replication or reactivation.

In contrast, the Iberian hare (*Lepus granatensis*) had been historically exposed to myxomatosis only sporadically [31] until the emergence of a recombinant MYXV strain capable of infecting hares, first detected shortly before the LeHV-5-associated disease outbreaks. The hare population, lacking prior exposure, displayed no immunological adaptation to MYXV, resulting in profound immunosuppression that likely facilitated LeHV-5 reactivation or exacerbation, leading to the observed herpetic lesions. This scenario highlights how an abrupt ecological and immunological disruption (a novel pathogen spillover) can modulate the clinical outcomes of latent viruses such as gammaherpesviruses.

In conclusion, the lack of pathological lesions in LeHV-6-infected rabbits likely reflects a combination of factors: the inherent biological behavior of gammaherpesviruses favoring latent infection in immunocompetent hosts, the long-standing co-evolution between rabbits and MYXV leading to limited immunosuppression, and the absence of an ecological perturbation analogous to the recent MYXV spillover in hares. Future studies employing histopathology, immunohistochemistry, and viral genome characterization will be essential to further explore the pathogenic potential of LeHV-6, particularly under conditions of immunosuppression or stress.

From an ecological perspective, understanding the prevalence, persistence, and pathogenic potential of LeHV-6 is vital. In a host species already burdened by emergent pathogens and habitat fragmentation, the additive or synergistic effects of latent herpesvirus infections may contribute to demographic instability. The role of LeHV-6 in wild rabbit health—whether as a silent passenger, a latent stress-activated pathogen, or an opportunistic co-infectant—remains an open question with important implications for conservation biology.

Beyond the phylogenetic evidence and the observed sequence divergence, the hypothesis that the identified herpesvirus represents a non-functional recombinant rather than a distinct viral entity appears highly unlikely from both molecular and ecological perspectives. Recombination in herpesviruses is indeed a well-documented phenomenon, but it predominantly occurs between strains circulating within the same host species or closely related lineages where co-infection is biologically plausible [5,12]. In this case, the distinct host specificity—Oryctolagus cuniculus for LeHV-6 versus Lepus granatensis for LeHV-5—combined with the lack of any evidence of LeHV-5 circulation in wild rabbit populations, strongly argues against a recent interspecies recombination event as the origin of LeHV-6. Additionally, the ecological separation between these host species limits opportunities for natural co-infection events that would be necessary to facilitate homologous recombination [32].

From a molecular standpoint, the genomic regions analyzed (DNA polymerase and glycoprotein B) are among the most conserved and functionally constrained in the herpesvirus genome [5,33]. These genes are essential for viral replication and structural integrity, and recombination events involving such core genes are extremely rare because they carry a high risk of generating non-viable or defective viral progeny [5,34]. Furthermore, recombination in herpesviruses generally affects regions associated with immune evasion, host adaptation, or latency rather than the replication machinery [5]. The relatively uniform divergence observed in both the DNA polymerase and gB genes supports a scenario of gradual evolutionary divergence rather than the mosaic pattern typically characteristic of recombinant genomes [35].

In addition, the hypothesis of a recombinant origin becomes even more unlikely considering that all sequences obtained from multiple individual animals were 100% identical in both DNA polymerase and gB genes. This genetic consistency strongly suggests the presence of a stable, circulating viral population rather than sporadic recombinant forms. If the virus were a defective or non-functional recombinant, a higher degree of sequence variability would be expected because of ongoing recombination or genetic instability. Instead, the identical sequences retrieved from different animals reinforce the conclusion that LeHV-6 represents a stable and distinct evolutionary lineage within the Gammaherpesvirinae, rather than the product of a recombination event between pre-existing viruses.

In summary, LeHV-6 represents a previously undescribed gammaherpesvirus circulating in Iberian wild rabbits. Its genetic and phylogenetic divergence from known leporid herpesviruses, coupled with ecological relevance and potential interactions with other pathogens, justify its designation as a novel species. Continued surveillance and genome-level characterization will be essential to assess its impact on host health and population dynamics.

## Figures and Tables

**Table 1:** List of primers used in this study.

Primer Name (F—Forward, R—Reverse)	Sequence (5′-3′)	Target	Reference
DFA (F, 1st round) *	GAYTTYGCNAGYYTNTAYCC	Herpesvirus *DNA* *Polymerase*	[10]
ILT (F, 1st round) *	TCCTGGACAAGCAGCARNYS GCNMTNAA		
KG1 (R, 1st round) *	GTCTTGCTCACC AGNTCNACNCCYTT		
TGV (F, 2nd round)	TGTAACTCGGTGTAYGGNTTYACNGGNGT		
IYG (R 2nd round)	CACAGAGTCCGTRTCNCCRTADAT		
2759s (F, 1st round) *	CCTCCCAGGTTCARTWYGCMTAYGA	HV *Glycoprotein B*	[12]
2762as (R, 1st round) *	CCGTTGAGGTTCTGAGTGTARTARTTRTAYTC		
2760s (F, 2nd round)	AAGATCAACCCCAC(N/I)AG(N/I)GT(N/I)ATG		
2761as (R 2nd round)	GTGTAGTAGTTGTACTCCCTRAACAT(N/I)GTYTC		
707s	TTGTGGACGAGRS(N/I)MAYTTYAT	HV *Terminase*	[13,14]
707as	ACAGCCACGCCNGT(N/I)CC(N/I)GA(N/I)GC		
708s	GCAAGATCATNTTYRT(N/I)TC(N/I)TC		
708as	TGTTGGTCGTRWA(N/I)GC(N/I)GGRT		
CSGdeg1F	GTIGAYGARRSIMAYTTYAT	HV *Terminase*	[15]
CSGdeg1R	TTKIIIGTRWAIGCIGGRTC		
CSGdeg2F	MYISYAARMTIATITTYRTITCITC		
CSGdeg2R	GTRWAIGCIGGRTCIAIRTA		
HV-TERM-F1	GTSAACTCWGGAGAMMAAWCYAC	HV *Terminase*	This study
HV-TERM-R1	GCRTCWCCCATTAATTCWGTWGT		
HV-TERM-F2	ATGCCARMGAMGACTYCTCAAT		
HV-TERM-R2	TCWCCCATWARCTCWGTTGTRAA		
HV-TERM-F3	CCCATTARTTCWGTKGYRAATG		
HV-TERM-R3	GTRAAYTCWGGWGARAARACAAC		
HV-TERM-F4	CCCATYARTTCTGTGGYRAARR		
HV-TERM-R4	GTIAATTCWGSAGAIAARICIACMAGT		
m000.5L/R F	CGACGTAGATTTATCGTATACC	MYXV Quadruplex	[16]
m000.5L/R	GTCTGTCTATGTATTCTATCTCC		
m000.5L/R P	[FAM]TCGGTCTATCCTCGGGCAGACATAGA[BHQ1]		
m009L F	TCCATTTACGATACACGCCGACGC		
m009L R	ACAACGTTCTATACTGTTTAGGGGGTACG		
m009L P	[TxRed]TACGATCTACTGACGAACGAATACAGTTTAATGCC[BHQ2]		
m060L F	GATTCTTTAATCTGGTTGAGGCAACTA		
m060L R	GGATATTATTACGCTCCATTATCGGAGG		
m060L P	[HEX] CTGATAAGTACCCCTTATCTACAAAAACGGGTG [BHQ1]		

* These primers were also used in the second round to allow longer amplicons.

## Data Availability

The nucleotide sequences generated during this study were deposited in the GenBank database under accession numbers PV730057 to PV730064. All other data supporting the findings of this study are available from the corresponding author upon reasonable request.
